# Supplementary material for: Causal Relationship between Aging and Anorexia Nervosa: A White-Matter-Microstructure-Mediated Mendelian Randomization Analysis
Source: Biomedicines. 2024 Aug 16;12(8):1874. doi: 10.3390/biomedicines12081874 (PMC11351342; doi:10.3390/biomedicines12081874)
Supplement: Supplementary file 1 [file biomedicines-12-01874-s001.zip › Supplementary Figure/Figure S4/Singe SNP plot of MR analysis for TL to ubm-b-1857.pdf]

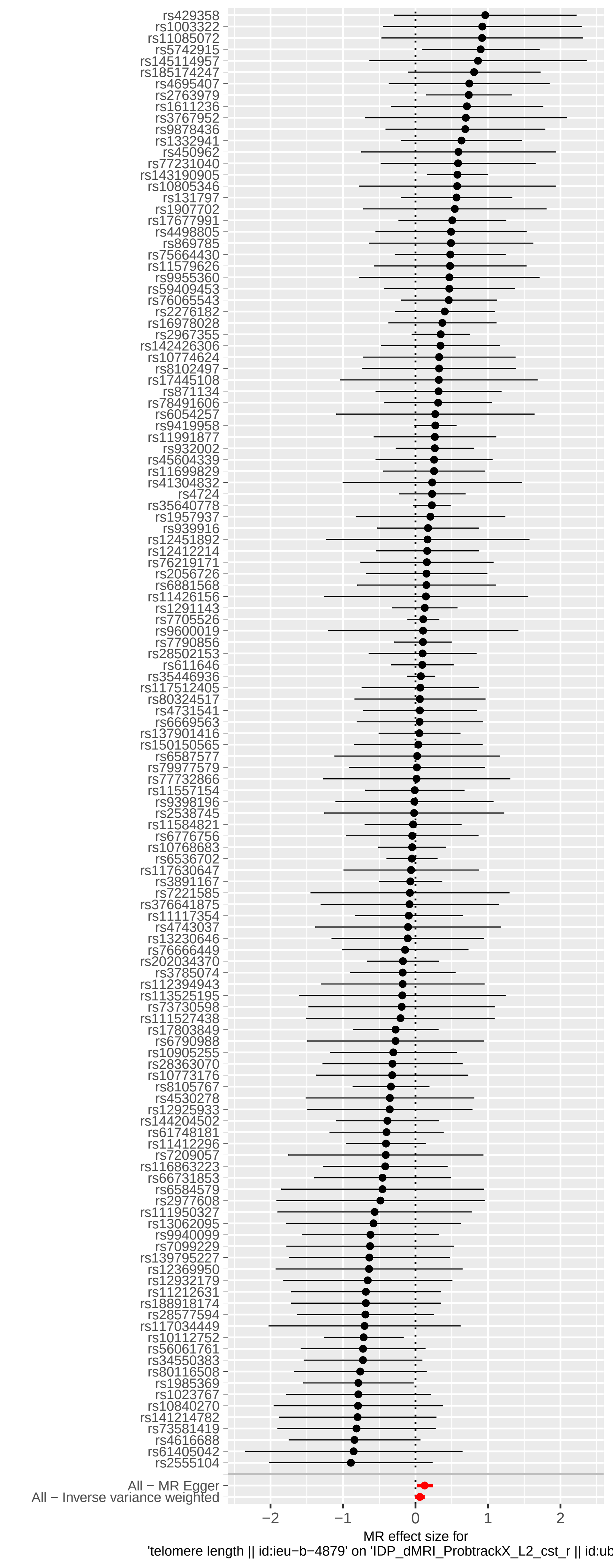
rs429358
rs1003322
rs11085072
rs5742915
rs145114957
rs185174247
rs4695407
rs2763979
rs1611236
rs3767952
rs9878436
rs1332941
rs450962
rs77231040
rs143190905
rs10805346
rs131797
rs1907702
rs17677991
rs4498805
rs869785
rs75664430
rs11579626
rs9955360
rs59409453
rs76065543
rs2276182
rs16978028
rs2967355
rs142426306
rs10774624
rs8102497
rs17445108
rs871134
rs78491606
rs6054257
rs9419958
rs11991877
rs932002
rs45604339
rs11699829
rs41304832
rs4724
rs35640778
rs1957937
rs939916
rs12451892
rs12412214
rs76219171
rs2056726
rs6881568
rs11426156
rs1291143
rs7705526
rs9600019
rs7790856
rs28502153
rs611646
rs35446936
rs117512405
rs80324517
rs4731541
rs6669563
rs137901416
rs150150565
rs6587577
rs79977579
rs77732866
rs11557154
rs9398196
rs2538745
rs11584821
rs6776756
rs10768683
rs6536702
rs117630647
rs3891167
rs7221585
rs376641875
rs11117354
rs4743037
rs13230646
rs76666449
rs202034370
rs3785074
rs112394943
rs113525195
rs73730598
rs111527438
rs17803849
rs6790988
rs10905255
rs28363070
rs10773176
rs8105767
rs4530278
rs12925933
rs144204502
rs61748181
rs11412296
rs7209057
rs116863223
rs66731853
rs6584579
rs2977608
rs111950327
rs13062095
rs9940099
rs7099229
rs139795227
rs12369950
rs12932179
rs11212631
rs188918174
rs28577594
rs117034449
rs10112752
rs56061761
rs34550383
rs80116508
rs1985369
rs1023767
rs10840270
rs141214782
rs73581419
rs4616688
rs61405042
rs2555104
All − MR Egger
All − Inverse variance weighted
−2
−1
0
1
2
MR effect size for
'telomere length || id:ieu−b−4879' on 'IDP_dMRI_ProbtrackX_L2_cst_r || id:ub
